# Alcoholic Beverage and Meal Choices for the Prevention of Noncommunicable Diseases: A Randomized Nutrigenomic Trial

**DOI:** 10.1155/2018/5461436

**Published:** 2018-06-27

**Authors:** Laura Di Renzo, Giorgia Cioccoloni, Paola Sinibaldi Salimei, Ida Ceravolo, Antonino De Lorenzo, Santo Gratteri

**Affiliations:** ^1^Clinical Nutrition and Nutrigenomic Section, Department of Biomedicine and Prevention, University of Rome Tor Vergata, Via Montpellier 1, 00136 Rome, Italy; ^2^PhD School of Applied Medical-Surgical Sciences, University of Rome Tor Vergata, Via Montpellier 1, 00133 Rome, Italy; ^3^Department of Experimental Biomedicine and Clinical Neuroscience, Ophthalmology Section, University of Palermo, Piazza Marina 61, 90133 Palermo, Italy; ^4^Department of Surgery and Medical Science, Magna Græcia University, 88100 Catanzaro, Germaneto, Italy

## Abstract

**Background:**

Noncommunicable diseases (NCDs) are the first cause of death worldwide. Mediterranean diet may play a crucial role in the prevention of NCDs, and the presence of wine in this diet could play a positive role on health.

**Methods:**

54 healthy volunteers consumed one of the following beverages: red (RW) or white wine (WW), vodka (VDK), and/or Mediterranean meal (MeDM) and high-fat meal (HFM).

**Results:**

OxLDL-C changed significantly between baseline versus HFM, MeDM versus HFM, and HFM versus HFM + RW (*p* < 0.05). Significant upregulation of catalase (CAT) was observed only after RW. Conversely, WW, VDK, RW + MeDM, HF + WW, and HF + VDK determined a significant downregulation of CAT gene. Superoxide dismutase 2 (SOD2) gene expression was upregulated in WW, MeDM + VDK, and RW. Contrariwise, HFM + VDK determined a downregulation of its expression. RW, RW + MeDM, and RW + HFM caused the upregulation of glutathione peroxidase-1 (GPX1).

**Conclusions:**

Our results suggest that the association of low/moderate intake of alcohol beverages, with nutraceutical-proven effectiveness, and ethanol, in association with a Mediterranean diet, could determine a reduction of atherosclerosis risk onset through a positive modulation of antioxidant gene expression helping in the prevention of inflammatory and oxidative damages.

## 1. Introduction

Noncommunicable diseases (NCDs) are the first cause of death worldwide. In 2011, the United Nations assembly recognized the social-economical-medical importance and the inevitability of preventive politics of NCDs in order to reduce the mortality indexes [1, 2], and in 2013, the World Health Organization (WHO) has laid down a policy paper for the institution and promotion of prevention politics in order to reduce the damages due to these pathologies. Cardiovascular diseases (CVDs) represent the 48% of NCDs, followed by cancer (21%), respiratory chronic diseases (12%), and diabetes (3.5%) [3, 4].

NCDs are the result of the individual predisposition, that is, genetic component, lifestyle habits, and pathological changes that lead to an untreatable full-blown chronic degenerative illness [5]. According to WHO, three of the most important behavioral risk factors for NCDs are harmful use of alcohol, unhealthy diets, which promote the progression and pathogenesis of polygenic diet-related diseases, and sedentary behavior [3].

The effect of some alcoholic beverages and dietary compounds on metabolic pathways related to several NCDs is currently under investigation and is leading the traditional methods of nutritional counseling towards a more complex approach based on the modulation of gene expression by food.

Wine is only the fourth (8.0%) most consumed among alcoholic beverage worldwide, and its consumption is higher in European regions (25.7%). In fact, globally, individuals above 15 years of age drink 13.5 g/day of pure alcohol, mainly in the form of spirits (50.1%) and beer (34.8%) [6]. It has been shown a relationship to “curved J-shaped” among the consumption of alcohol and the mortality, in which harmful effects are reached after 89 g of average intake for day and a maximum protective effect to 20 g of pure average alcohol intake per day [7].

If on one side, the toxic role of the consumption of alcoholic beverages is broadly shown, associated with an increased overall mortality, cardiomyopathy, hypertension, acute cerebrovascular events, liver diseases, and cancer, and on the other side, several epidemiologic studies have encountered an inverse association between risk of cardiovascular mortality and moderate alcohol consumption [8]. It is likely that the cardioprotective effect of alcohol is due to its ability to increase high-density lipoprotein-cholesterol (HDL-C) levels and its antithrombotic properties.

Moreover, wine represents an important component of the Mediterranean diet (MeD), contributing to the reduced incidence of NCDs of this dietary habit [9]. Important evidences about a likely positive relation between nutrition and cardiovascular disorders were showed thanks to epidemiological studies on Greek and southern Italian people, in which the incidence of CVDs was remarkably low compared with other regions of the world [9, 10]. In Italy, even today, wine is the favorite beverage among adults [11].

Among alcoholic beverages, wines, especially red ones, could play a positive role against CVDs, as observed in PREDIMED study [12]. For instance, this beverage, thanks to the resveratrol and polyphenolic compounds, may decrease blood viscosity, antagonize the development of endothelial dysfunction increasing nitric oxide (NO) bioavailability, and reduce atherosclerosis by inhibiting lipoprotein oxidation and thrombosis [13].

Considering the remarkable influence of red wine on CVDs, in further researches, it has been analyzed the possibility that other alcoholic beverages could have the same influence. For the time being, some interesting results were showed for white wine. This beverage, which contains phenols like red wine and olive oil, seems to have a good effect in preventing inflammatory cytokine release and it may also play an interesting role in the cardioprotection [14]. On the contrary, for spirits, conflicting data are demonstrated. For instance, vodka may have good capability to prevent both hyperoxia-induced increase of arterial stiffness [15] and endothelial dysfunction, reducing oxidative stress in the myocardium [16]. In other studies data for this beverage are not encouraging [17].

Another important aspect for the prevention of NCDs is the type and quality of food. It is widely accepted that the consumption of fruits and vegetables prevents diseases related to the oxidative processes [9]. Conversely, one of the common causes of oxidative stress onset, and then of the vascular alterations of NCDs, is the huge consumption of salt, sugars, and processed meals and a low intake of vegetables and fruit [18].

Further researches demonstrated that MeD might play a crucial role in the prevention of NCDs, because of the high content of polyunsatured fatty acids (PUFA), polyphenols, and fiber and the low content of saturated fatty acids, cholesterol, and sodium [9, 19–21].

We have previously demonstrated that red wine in association with McDonald's® and a Mediterranean meal modulated the inflammatory status and could represent an essential component of a holistic approach to combatting chronic NCDs linked to inflammation [22].

Based on previous results, we hypothesized that 30 g of ethanol and polyphenol present in different types of alcoholic beverages, that is, red wine, white wine, and vodka, could amplify the effect of an antioxidant meal and lead to a change in oxidative status, contributing to a reduction of chronic NCDs linked to inflammation [23]. The first endpoint of this study was to examine the oxidative status of LDL; the second end point was the evaluation of gene expression of selected genes belonging to inflammatory and oxidative stress pathway, as catalase (CAT), superoxide dismutase 2 (SOD2), and glutathione peroxidase 1 (GPX1). Therefore, a controlled randomized clinical trial was performed on healthy volunteers in fasting status or in the postprandial time, after a Mediterranean or a high-fat meal, with or without alcoholic beverages intake.

## 2. Materials and Methods

### 2.1. Subjects and Study Design

**Figure 1:**
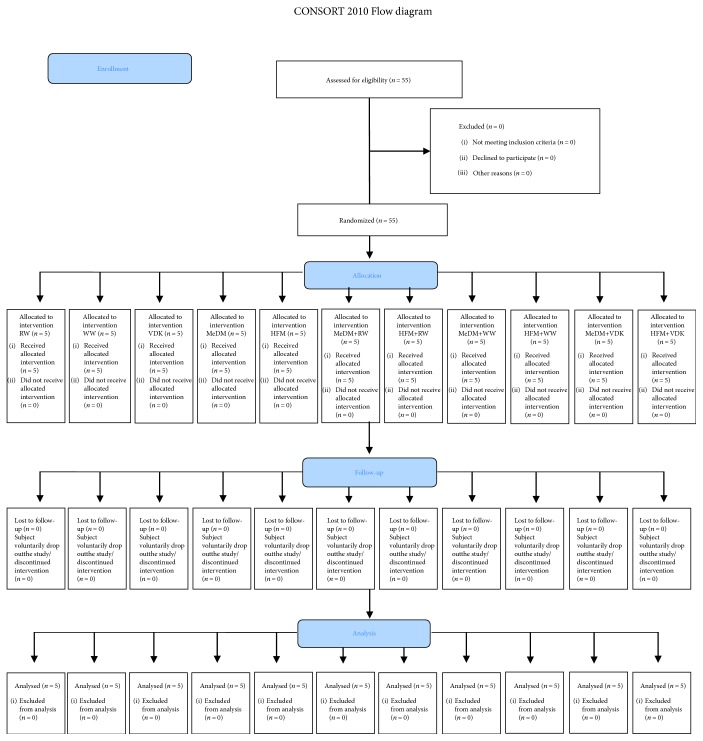
Study design. Clinical trial design. RW: fasting red wine; WW: fasting white wine; VDK: fasting vodka; MeDM: Mediterranean meal; HFM: high-fat meal; MeDM + RW: Mediterranean meal plus red wine; MeDM + WW: Mediterranean meal plus white wine; MeDM + VDK: Mediterranean meal plus vodka; HFM + RW: high-fat meal plus red wine; HFM + WW: high-fat meal plus white wine; HFM + VDK: high-fat meal plus vodka.

For this study, 55 healthy volunteers were recruited at the Clinical Nutrition and Nutrigenomic Section at the University of Rome Tor Vergata. In order to be included in the study, subjects had to respect the following eligibility criteria: age between 18 and 65 years old and BMI between 18.5 and 35 kg/m^2^. At the same time, exclusion criteria were the following: BMI > 35 kg/m^2^, active tobacco smoking, past or active cardiovascular, hepatic, metabolic, autoimmune, and neoplastic diseases, and drug consumption. The randomized parallel group study was conducted as shown in Figure 1. Blood samples for oxLDL-C concentration and biochemical and genomic analyses were collected at baseline (B), and genomic analysis also after 2 h of intervention in order to evaluate nutritional, oxidative, and inflammation status. Nutritional status assessment and genomic analysis were performed at the Clinical Nutrition and Nutrigenomic Section, Department of Biomedicine and Prevention of University of Rome Tor Vergata. Lifestyle habits of healthy volunteers did not change during the study period. Clinicians assessed any adverse effects from the interventions by going through a checklist of symptoms, including bloating, fullness, or indigestion, altered bowel habit, dizziness, and other symptoms that were possibly associated with the interventions. No abnormality was presented during the study period. All patients completed the study. All participants, in accordance with principles of the Declaration of Helsinki, signed a statement of informed consent. This protocol has been registered with ClinicalTrials.gov NCT01890070.

### 2.2. Dietary Intervention

During the study, subjects randomly consumed one of the following beverages or meals: (a) fasting + 30 g of ethanol from red wine (RW), (b) fasting + 30 g of ethanol from white wine (WW), (c) fasting + 30 g of ethanol from vodka (VDK), (d) Mediterranean meal (MeDM), (e) high-fat meal (HFM), (f) MeDM + 30 g of ethanol from RW, (g) MeDM + 30 g of ethanol from WW, (h) MeDM + 30 g of ethanol from VDK, (i) HFM + 30 g of ethanol from RW, (j) HFM + 30 g of ethanol from WW, and (k) HFM + 30 g of ethanol from VDK. MeMD was composed by 150 g of whole pasta, 300 g of eggplants, 150 g of tomatoes, 150 g of peppers, 100 g of rocket salad, 100 g of radicchio, 60 g of anchovies, 20 g of walnuts, and 20 g of capers. The HFM was bought in McDonald's restaurant and was represented by n.1 Big Tasty Bacon® and n.1 small French fries package. The MeDM was prepared, distributed, and consumed as well as HFM, at the Clinical Nutrition and Nutrigenomic Section, Department of Biomedicine and Prevention, University of Rome Tor Vergata. Meal analyses were performed by the Dietosystem dietary software (DS Medica S.r.l., Milan, Italy).

RW, made from Merlot (75%), Tocai Rosso (10%), and Cabernet Sauvignon (15%) grapes, was used in the study. RW characteristics are as follows: unfiltered wine, no added sulfites, total alcohol: 14.52% volume, residual sugar: 0.7 g/l, total acidity: 5.9 g/l, dry extract: 30 g/l, volatile acidity: 0.59 g/l, and total sulfur dioxide: 2 mg/l. WW made from Garganega grapes (95%) and other variety (5%) was used in the study. WW characteristics are as follows: unfiltered wine, no added sulfites, total alcohol: 12.5% volume, residual sugar: 0.6 g/l, total acidity: 4.7 g/l, dry extract: 18 g/l, volatile acidity: 0.48 g/l, and total sulfur dioxide: 9 mg/l. The commercial vodka used in this study was a pure grain distilled with total alcohol volume of 38%.

Subjects were not blinded to the type of beverages or meal they consumed.

### 2.3. Anthropometric Measurements

All volunteers were subjected to anthropometric evaluation after overnight fasting. Body weight and height were measured according to standard procedures [20]. Body weight was evaluated with balance scale to the nearest 0.1 kg (Invernizzi, Rome, Italy). Height was measured with a stadiometer to the nearest 0.1 cm (Invernizzi, Rome, Italy). BMI was calculated using the formula: BMI = body weight (kg)/height (m^2^).

### 2.4. Biochemical Analysis

Blood tests were carried out at the accredited Clinical Chemical Laboratories of the “Policlinic Tor Vergata (PTV)” of Rome, Italy. Analyses were performed only at baseline after a 12-hour overnight fast. Blood samples (10 ml) were collected into tubes with EDTA (Vacutainer®). Samples were directly placed on ice, and plasma was separated by centrifugation.

Laboratory analysis included complete blood count, fasting glucose, total cholesterol (TC), HDL-C, LDL-cholesterol (LDL-C), triglycerides (Tg), aspartate aminotransferase (AST/GOT), alanine transaminase (ALT/GPT), fibrinogen, albumin, creatinine, erythrocyte sedimentation rate (ESR), C-reactive protein (CRP), and insulin. All clinical chemistry analyses, except fasting glucose, serum lipid, CRP, and Tg analyses, were carried out with an ADVIA®1800 Chemistry System (Siemens Healthcare). Plasma glucose concentrations were measured with glucose oxidase method (COBAS INTEGRA 400, Roche Diagnostics, Indianapolis, IN, USA); serum lipid profile components were determined by standard enzymatic colorimetric techniques (Roche143 Modular P800, Roche Diagnostics, Indianapolis, IN, USA). Serum Tg was measured by a coupled enzymatic method on the Beckman Synchron LX20 automated system. Serum CRP was measured by a high-sensitivity sandwich enzyme immunoassay (Immundiagnostik, Bensheim, Germany). In order to minimize variability, all tests were performed using the same lot of reagents or assay plates.

The assessment of insulin resistance was evaluated with the homeostasis model assessment of insulin resistance (HOMA-IR) corresponding to the following formula:

(1) $$\begin{array}{c} HOMA‐IR=\frac{fasting\;glucose\left(mg/dl\right)\times fasting\;insulin\left(uU/ml\right)}{405}. \end{array}$$

### 2.5. Sample Collection and RNA Extraction

Blood samples were collected in PAXgene Blood RNA Tubes (PreAnalytiX Qiagen, Hombrechtikon, Switzerland) and stored at −80°C until use. RNA from each sample was extracted with PAXgene Blood miRNA Kit (PreAnalytix Qiagen, Hombrechtikon, Switzerland) according to the manufacturer's instructions. RNA quantification was performed through spectrophotometry (NanoDrop, Wilmington, USA).

### 2.6. Quantitative Real-Time PCR and Data Analysis

For the assessment of SOD2 (NCBI Reference Sequence: NC_000006.12), CAT (NCBI Reference Sequence: NC_000011.10), and GPx1 (NCBI Reference Sequence: NC_000003.12) genes, the human oxidative stress (PAHS-065ZA) and the inflammatory cytokine and receptor (PAHS-011Z) RT^2^ Profiler PCR Arrays (Qiagen, Netherlands) were used. Each sample was analyzed in triplicate and repeated twice according to the manufacturer's instructions (Qiagen, Netherlands). We used *β*-actin (ACTB) (NM 001101) as a housekeeping gene. To determine gene expression level, comparative threshold (CT) cycle was used. CT value was normalized using the formula ΔCT = CT (gene) − CT (housekeeping gene). The relative gene expression levels were determined according to the following formula: ΔΔCT = ΔCT sample − ΔCT calibrator. The value used to plot relative gene expression was determined using the expression fold change (FC) = 2^(−ΔΔCT)^.

### 2.7. Low-Density Lipoprotein Oxidative Status

Blood samples were collected, stabilized, and centrifuged in EDTA tubes (Vacutainer). Plasma was removed and stored at −80°C until use. Mercodia Oxidized LDL ELISA test (Mercodia AB, Sweden) was used to quantify oxLDL-C according to the manufacturer's protocol. All the analyses were performed in triplicate.

### 2.8. Statistical Analysis

Statistical analysis was carried out using IBM SPSS 21.0 for Windows (IBM Corp., Armonk, NY, USA). After the Shapiro-Wilk test, a paired *t*-test or a nonparametric Wilcoxon test was performed to evaluate differences before and after nutritional interventions. Parametric *t*-test or nonparametric Mann–Whitney *U* test was performed to evaluate differences between nutritional interventions. All tests were considered significant at *p* ≤ 0.05. Percentage variations were calculated as the percentage difference between baseline and the subsequent treatment. For genomic analysis, the value used to plot relative gene expression was determined using the expression fold change (FC) = 2^(−ΔΔCT)^, using *β*-actin (ACTB) as housekeeping gene. Only genes with a FC ≥ 2 were considered significant upregulated for differentially expressed genes. Conversely, genes with a FC ≤ 0.5 were considered significant downregulated for differentially expressed genes.

## 3. Results

### 3.1. Meal Analysis

MeDM was composed of 55% of carbohydrates, 20% of proteins (>50% of vegetable derivation), <30% of lipids (on total kcal: saturated fat < 10%, 6–10% polyunsaturated fatty acids (PUFA), n-6 : n-3 PUFA ratio of 3 : 1, 15% of monounsaturated fatty acids (MUFA), and <1% trans-fatty acids), and 30 g of fiber. HM was composed by 24.3% of carbohydrates, 23% of proteins (>80% animal proteins), 52% of total fat (saturated fat 19.5% of total kcal), and 5.60 g of fiber. Furthermore, MeDM had the following nutritional index values: cholesterol-saturated fat index (CSI) of 8.80, thrombogenic index (TI) of 0.46, atherogenic index (AI) of 0.26, and potential renal acid load (PRAL) of −23.56. At the same time, HF meal had the following nutritional index values: CSI of 24.34, TI of 1.73, AI of 1.97, and PRAL of 29.57.

### 3.2. Subject Characteristics

Of the fifty-five subjects enrolled, one of them was excluded from the trial (subject declined to participate). Finally, fifty-four patients completed the study (Figure 1). No changes to trial outcomes after the trial commenced occurred. The average age of subjects was 32.47 ± 7.25 years, 58.8% females and 41.2% males; none of the subjects presented metabolic diseases (Table 1).

### 3.3. OxLDL-C Analysis

**Figure 2:**
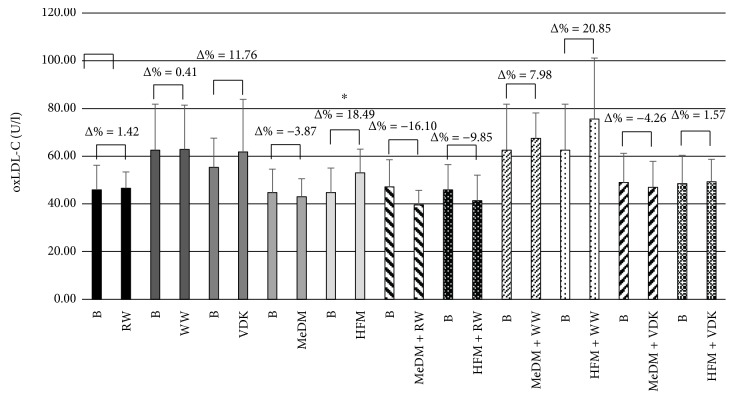
Variation of oxLDL-C levels between baseline and treatments. Comparative values of oxLDL-C levels for each treatment intervention. The significant values are expressed as B versus HFM (^∗^ *p* < 0.05). B: baseline; RW: fasting red wine; WW: fasting white wine; VDK: fasting vodka; MeDM: Mediterranean meal; HFM: high-fat meal; MeDM + RW: Mediterranean meal plus red wine; MeDM + WW: Mediterranean meal plus white wine; MeDM + VDK: Mediterranean meal plus vodka; HFM + RW: high-fat meal plus red wine; HFM + WW: high-fat meal plus white wine; HFM + VDK: high-fat meal plus vodka.

Comparing oxLDL-C levels at baseline and after the consumption of different beverage and/or meal treatments, significant changes were observed only between baseline and HFM treatment (Δ% = 18.49; *p* < 0.05) (Figure 2). Among treatments, we noticed significant differences in oxLDL-C levels between MeDM and HFM (*p* < 0.05) and HFM and HFM + RW (*p* < 0.05) (Table 2). Conversely, no statistical significant modifications between beverages treatments, MeDM meals associated with RW, WW, and VDK consumption as well as among HFM and WW and VDK administrations were determined.

### 3.4. Gene Expression Data

**Figure 3:**
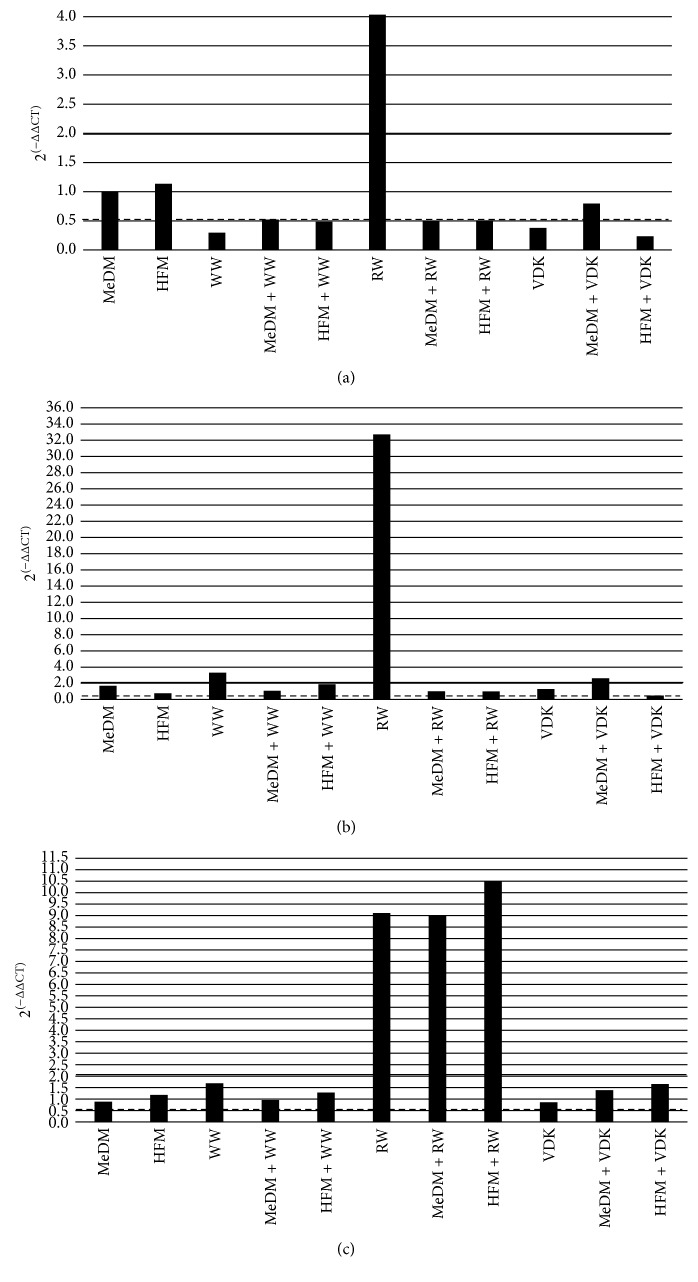
Gene expression after treatments. Different levels of fold change of genes analyzed: (a) CAT: catalase, (b) SOD2: superoxide dismutase 2, and (c) GPX1: glutathione peroxidase 1. RW: fasting red wine; WW: fasting white wine; VDK: fasting vodka; MeDM: Mediterranean meal; HFM: high-fat meal; MeDM + RW: Mediterranean meal plus red wine; MeDM + WW: Mediterranean meal plus white wine; MeDM + VDK: Mediterranean meal plus vodka; HFM + RW: high-fat meal plus red wine; HFM + WW: high-fat meal plus white wine; HFM + VDK: high-fat meal plus vodka.

Significant upregulation of CAT, with a fold change exceeding the threshold set at 2, was observed only after RW (2^(−ΔΔCT)^ = 4.04). Conversely, WW and VDK administration determined a significant downregulation of CAT gene expression (2^(−ΔΔCT)^ = 0.30 and 2^(−ΔΔCT)^ = 0.23, resp.) as well as the combination of HFM with WW (2^(−ΔΔCT)^ = 0.48) and VDK (2^(−ΔΔCT)^ = 0.23) (Figure 3(a)). The expression of SOD2 gene was upregulated in WW, MeDM + VDK treatment, and especially in RW administration (2^(−ΔΔCT)^ = 3.32, 2^(−ΔΔCT)^ = 2.63, and 2^(−ΔΔCT)^ = 32.73, resp.). On the other hand, HFM + VDK treatment determined a downregulation of its expression (2^(−ΔΔCT)^ = 0.49) (Figure 3(b)). RW alone and its association with MeDM and HFM treatments caused the upregulation of GPX1 gene expression (2^(−ΔΔCT)^ = 9.12, 2^(−ΔΔCT)^ = 8.99, and 2^(−ΔΔCT)^ = 10.5, resp.) (Figure 3(c)).

## 4. Discussion

NCDs share lifestyle as a risk factor. It seems particularly important to consider patient living conditions, before that NCDs lead to an untreatable full-blown chronic degenerative illness. In fact, lifestyle habits could make a difference in normal and pathological conditions. Primary prevention starts essentially from small lifestyle changes. Actually, in 2012, the Medical American Association invited health professionals to apply the “lifestyle medicine” clinical skills as a NCDs primary prevention [24].

One of the best known biochemical mechanisms underlying aging is the progressive loss by senescent cells of perfect replication of DNA in daughter cells. The “errors” accumulated in the transcription of cellular DNA after several replication processes end up by activating particular gene sequences which are self-replicating and which accumulate within the cell over time, causing its degeneration and death. The formation of these “cell scars” is closely related to aging. The possibility of reducing accumulated errors, modulating the inflammatory processes that underlie them, is the subject of numerous studies that focus on nutraceutical aspects and the concept of quality.

The glucose and lipid hematic concentrations in postprandial period, as the increased concentration of LDL-C converted in their oxidize form, the oxidized low-density lipoprotein-cholesterol (oxLDL-C), lipoxygenases, and the reactive oxygen species (ROS) [25], were related to the chronic process pathogenesis of NCDs [26].

ROS levels raise in postprandial state damaging cellular structures and contributing to the activation of some transcription factors that are able to regulate the expression of genes involved in immunity, inflammation, cell proliferation, growth, and apoptosis [27, 28]. OxLDL-C accumulates in the tunica intima where engulfed by macrophages, thereby formation of cholesterol laden foam cells, which is considered the initial event of atherosclerosis [16]. Furthermore, the inflammation induced by oxidative stress and oxLDL-C on vascular cells increases monocyte and macrophage adhesion and infiltration into the vessel wall causing the foam cell development [29, 30]. In this frame, antioxidant enzymes like SODs, GPX1, and CAT take on great importance in the reduction of circulating ROS as well as in LDL oxidation process. In fact, superoxide anion (O_2_^−^), which belongs to the free radical molecules of ROS family, can be converted naturally or enzymatically by SODs into hydrogen peroxide (H_2_O_2_), which, in turn, can be transformed into water by CAT or GPX1 [31]. SOD2, through the control of mitochondrial ROS and NO, regulates endothelial and vascular smooth muscle cells. Its deficiency seems to be involved in onset and development of atherosclerosis through the increase of O_2_^−^ levels and mitochondrial dysfunction, which leads to mitochondrial DNA damage [32]. CAT, instead, can react with H_2_O_2_ through the four groups of porphyrin heme iron, speeding up its conversion into water. It was observed that reduced expression of CAT can induce atherosclerosis onset and progression [33]. GPX1 is one of the most represented enzymes of GPX family and its ability to reduce H_2_O_2_ into water makes it inversely associated with CVD risk [34]. In fact, GPX1 shortage determines increasing foam cell formation by oxLDL-C, enhancing atherosclerosis process [35].

For many years, the benefits of fruits and vegetables have long been considered due to their fibers, minerals, and vitamin contents, but studies made in the last few decades have highlighted the importance of phytochemicals in preventing disease and increasing life expectancy [36]. The life expectancy of humans can be prolonged by an appropriate diet, rich in vegetables and fruit, which contain antioxidants. Foodstuffs with a relevant antioxidant effect do contain molecules that can prevent damage to the cellular system incurred by ROS [37].

Novel dietary strategies provide a new window of opportunity in the efforts being made to reduce NCDs and numerous so-called functional foods being proposed for the promotion of health, with simple lifestyle changing. From an evolutionistic point of view, our genes are evolved for metabolizing fermented plant materials, so that the moderate consumption of wine became physiologically an integral part of our diet.

The preventive role of the moderate consumption of wine has broadly been shown and envoy in relationship to the content of phenolic substances [38], mainly resveratrol and proanthocyanidins and other polyphenols (mostly present in red wine) including flavonols, monomeric flavan-3-ols, and anthocyanin [39–41]. The polyphenols, particularly resveratrol and quercetin, contribute to prevent or delay the onset of chronic diseases such as diabetes, inflammation, Alzheimer's disease, and cardiovascular disease. Moreover, resveratrol induces neuroprotection and inhibits proliferation of human cancer cell lines [42–46].

Both resveratrol and quercetin are able to reduce ROS concentrations in different tissues [44–46], and their dietary supplementation increases CAT, SOD, and GPX expression [47–49]. Thanks to its high concentrations of polyphenols, RW consumption reduces LDL oxidation and prevents endothelial dysfunction [23]. Conversely, WW exhibits lower antioxidant capacity reducing LDL oxidation when compared to RW [50]. Furthermore, WW in rat and human vascular smooth muscle cells has no effect on the development of atherosclerosis [51]. A possible explanation is the low polyphenolic content of WW compared to RW [52] due to the lack of expression of the enzyme flavonoid 3′,5′-hydroxylase in white grapes, which restricts the presence of flavonol and anthocyanin contents [53]. These evidences support the common thought that polyphenols are the reason why wine has beneficial effect on CVD prevention, independently from ethanol content. In fact, dealcoholized RW but not WW greatly increased in vitro plasma antioxidant capacity, reduced oxLDL-C concentrations, improved flow-mediated vasodilation, and increased human endothelial NO synthase [54, 55]. However, data on the role of ethanol and spirts are controversial. Recently, Fawole and Opara [56], throughout an in vitro digestion model, demonstrated that the total phenolic concentration and total flavonoid concentration of a food rich in polyphenols are influenced by the extraction solvents used. In particular, the polyphenol bioavailability is higher in ethanol solvent.

VDK decreases protein oxidative stress in the myocardium but has no effect on normalizing endothelial dysfunction and platelet aggregation [16, 57, 58]. However, VDH seems to not exert protection against oxygen-induced oxidative stress in plasma lipid peroxides [59].

**Figure 4:**
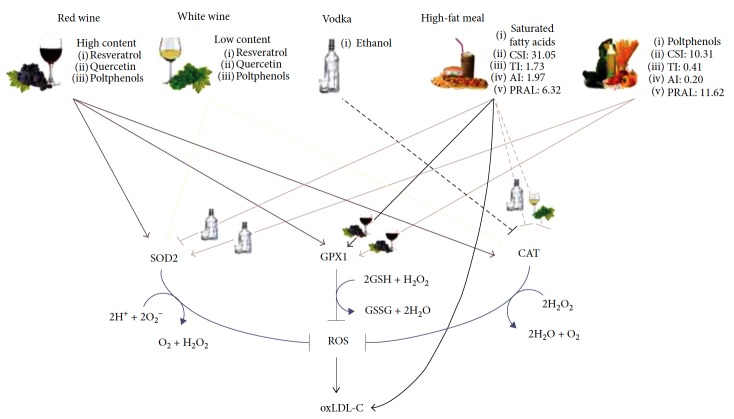
Effects of beverage and/or meal administration on oxLDL-C and gene expression. Effects of fasting red wine, fasting white wine, fasting vodka, Mediterranean meal, high-fat meal, Mediterranean meal plus red wine, Mediterranean meal plus white wine, Mediterranean meal plus vodka, high-fat meal plus red wine, high-fat meal plus white wine and high-fat meal plus vodka on catalase (CAT), superoxide dismutase 2 (SOD2), glutathione peroxidase 1 (GPX1), and oxidized low-density lipoprotein-cholesterol (oxLDL-C).

According to previous studies [55, 60], we did not observe significant changes in oxLDL-C concentrations following RW, WW, and VDK consumption as well as among beverage treatments. WW and VDK administrations determined a reduction of CAT expression (2^(−ΔΔCT)^ = 0.30 and 2^(−ΔΔCT)^ = 0.23, resp.), suggesting a possible downstream reduction of antioxidant enzyme gene expression caused by alcohol with low or null polyphenolic content. On the other hand, in WW and RW treatments, but not after VDK consumption, we observed an upregulation of SOD2 (2^(−ΔΔCT)^ = 3.32 and 2^(−ΔΔCT)^ = 32.73, resp.), suggesting a possible greater sensitivity of this gene to both high and low polyphenol concentrations, as reported also by Zhao et al. [60]. At the same time, RW consumption, beyond the increased levels of SOD2, determined an upregulation of CAT and GPx1 expression (2^(−ΔΔCT)^ = 4.04 and 2^(−ΔΔCT)^ = 9.12, resp.) (Figure 4), demonstrating that there is an acute indirect antioxidant response to RW polyphenolic content. However, the fast antioxidant activity exerted by RW did not highlight any changes in oxLDL-C levels. This result is probably due to the little time of plasma exposure to the RW, as also observed by Caccetta et al. [61].

Like RW, Mediterranean diet has been associated with a reduction of coronary events and CVD risk and mortality, probably due to the high content of antioxidants which are contained in the most represented foods of this dietary pattern, that is, vegetables, fruits, legumes, grains, nuts and seeds, fish, and wine [62–65]. Previous studies demonstrated the inverse correlation between Mediterranean diet and plasma oxLDL-C levels [66] as well as the relationship between this diet and antioxidant genes [22]. Conversely, one of the most important risk factors for CVD onset is the regular consumption of a high-fat diet. High-fat meals usually determine increased levels of oxidative stress and endothelial impairing in postprandial period. This process is mainly due to the high presence of saturated fatty acids in meals, which determine a transient hypertriglyceridemia that can activate mitochondrial metabolism and consequently enhance ROS production, leading to oxidative stress and/or lower antioxidant defenses and vascular damage [66]. According to our previous study [22], MeDM administration, with or without beverage combinations, did not determine a significant reduction of oxLDL-C levels compared to baseline as well as between MeDM alone and its association with beverages. A significant reduction of oxLDL-C levels was predictably observed between HFM and MeDM (*p* < 0.05). This effect is probably due to the different polyphenolic and antioxidants intake among meals [22] as well as the increasing levels of oxLDL-C among HFM and baseline (Δ% = + 18.49; *p* < 0.05), depending on the high presence of saturated fatty acids in the meal, which are able to enhance oxidative stress and reduce antioxidant defenses [66].

However, we noticed an upregulation of SOD2 expression (2^(−ΔΔCT)^ = 2.63) after MeDM + VDK administration, increased GPX1 gene expression in MeDM + RW (2^(−ΔΔCT)^ = 8.99) and HFM + RW group (2^(−ΔΔCT)^ = 10.5), and the upregulation of all antioxidant genes observed after the ingestion of RW, acknowledging the role of the antioxidant molecules present in RW on oxidative stress. Conversely, HFM treatment in association with VDK reduced both CAT and SOD2 expressions (2^(−ΔΔCT)^ = 0.23 and 2^(−ΔΔCT)^ = 0.49, resp.), as its combination with WW administration downregulated CAT (2^(−ΔΔCT)^ = 0.48). According to Fawole and Opara [56], these results, together with the reduction of oxLDL-C levels observed after HFM + RW treatment compared to HFM (*p* < 0.05) (Figure 4), suggest a pivotal role of ethanol on the bioavailability of polyphenols during digestion.

## 5. Conclusions

In order to maintain a good health status, it is necessary to have good nutritional habits. Food provides substances that can affect internal homeostasis and then lead to an untreatable full-blown chronic degenerative illness. Our findings support, for the first time based on nutrigenomic approach, the evidence that moderate alcohol consumption has significant health benefits, justifying the promotion of longevity and reduction of the risks of most of the age-related diseases. However, our data should be confirmed on a larger number of subjects, with a prospective long-term trial.

In this work, we observed that genetic regulation due to red wine consumption occurs both with the beverage alone and in combination with a meal, resulting as a protective food in postprandial state mainly because of its polyphenolic content, which is activated by alcohol. On the other hand, ethanol has a positive effect on gene oxidation pathway only if combined with an antioxidant meal, exerting a potential increase of polyphenols bioavailability during digestion and antioxidant genes expression, controlling LDL-C oxidation pathway.

Within a comprehensive vision of lifestyle medicine [67], according to other studies [68], our results suggest that the association of low/moderate intake of alcohol beverages with nutraceutical-proven effectiveness and ethanol in association with a Mediterranean diet could determine a reduction of atherosclerosis risk onset maintaining postprandial oxLDL-C levels steady and a positive modulation of antioxidant gene expression. Moreover, we highlighted the importance to choose healthy meals associated to alcoholic beverages for the prevention of inflammatory and oxidative damages.

In conclusion, we suggest that a good dietetic plan, finalized to the reduction of NCDs onset and progression, should contemplate a moderate consumption of alcoholic beverages.

## Figures and Tables

**Table 1:** Anthropometric and bioclinical baseline characteristic of study subjects.

Baseline characteristic of volunteers		
Parameters	Median ± SE	Min–Max
Age (y)	32.47 ± 7.25	25.00–52.00
Weight (kg)	65.11 ± 12.21	48.00–92.70
Height (cm)	168.06 ± 11.89	150.00–186.00
BMI (kg/m^2^)	22.98 ± 3.15	18.75–32.00
WBC (K/*μ*l)	6.51 ± 2.90	3.30–13.40
LYM (K/*μ*l)	1.96 ± 0.55	1.30–3.30
MON (K/*μ*l)	0.35 ± 0.13	0.20–0.60
GRN (K/*μ*l)	4.12 ± 2.36	1.70–10.30
EOS (K/*μ*l)	0.09 ± 0.08	0.00–0.20
BAS (K/*μ*l)	0.02 ± 0.06	0.00–0.20
LYM (%)	28.83 ± 10.18	1.84–41.00
MON (%)	5.03 ± 1.92	0.37–8.00
GRN (%)	57.00 ± 17.92	1.96–77.00
EOS (%)	1.61 ± 1.45	0.00–5.00
BAS (%)	0.19 ± 0.38	0.00–1.00
RBC (M/*μ*l)	4.62 ± 0.31	4.02–4.95
HGB (g/dl)	13.84 ± 1.26	11.10–16.50
HCT (%)	42.25 ± 2.98	36.90–48.20
MCV (fl)	91.51 ± 4.97	81.00–100.00
MCH (pg)	29.98 ± 2.55	24.30–34.70
MCHC (g/dl)	32.71 ± 1.38	30.20–17.90
RDW (%)	15.31 ± 1.63	13.00–17.90
PLT (K/*μ*l)	225.54 ± 78.24	135.00–438.00
PCT (%)	0.15 ± 0.02	0.12–0.19
MPV (fl)	7.30 ± 0.60	6.10–8.10
PDW (%)	51.30 ± 3.88	44.40–55.80
TC (mg/dl)	174.43 ± 28.86	128.00–233.00
HDL-C (mg/dl)	50.77 ± 10.21	38.00–69.00
LDL-C (mg/dl)	99.00 ± 25.01	51.00–137.00
Tg (mg/dl)	67.85 ± 28.16	35.00–120.00
Glycemia (mg/dl)	84.57 ± 10.10	65.00–101.00
Fibrinogen (mg/dl)	328.82 ± 96.84	226.00–480.00
GOT (U/l)	25.15 ± 11.23	10.00–56.00
GPT (U/l)	22.61 ± 7.52	10.00–38.00
CPR (mg/dl)	0.51 ± 0.51	0.10–1.72
ESR (mm/h)	8.85 ± 4.45	3.00–17.00
Insulin (*μ*M/ml)	6.82 ± 1.68	5.00–8.00
HOMA-IR	1.26 ± 0.31	0.83–1.63
Albumin (g/dl)	3.83 ± 0.33	3.50–4.31
Creatinine (mg/dl)	0.70 ± 1.14	0.92–0.16

Results are expressed in median ± standard error and minimum and maximum for each parameter. ALT/GPT: alanine transaminase; AST/GOT: aspartate aminotransferase; BAS: basophiles; CRP: C-reactive protein; EOS: eosinophils; ESR: erythrocyte sedimentation rate; GRN: granulocytes; HDL-C: high-density lipoprotein cholesterol; HCT: hematocrit; HGB: hemoglobin; HOMA-IR: homeostasis model assessment of insulin resistance; LDL-C: low-density lipoprotein cholesterol; LYM: lymphocytes; MCH: mean corpuscular hemoglobin; MCHC: mean corpuscular hemoglobin concentration; MCV: mean corpuscular volume; MPV: mean platelet volume; MON: monocytes; PDW: platelet distribution width; PLT: platelets; PCT: procalcitonin; RBC: red blood cells; RDW: red cell distribution width; TC: total cholesterol; Tg: triglycerides; WBC: white blood cells.

**Table 2:** OxLDL-C percentage variation between baseline and dietary treatment.

OxLDL-C percentage variation		
	Mean ± SD	Median (Min–Max)
Δ% B-RW	6.99 ± 23.96	11.3 (−39–48)
Δ% B-WW	1.21 ± 8.50	4.08 (−13–10)
Δ% B-VDK	9.76 ± 19.96	9.05 (−11–32)
Δ% B-MeDM	−1.32 ± 20.43^a^	3.66 (−43–47)
Δ% B-HFM	21.29 ± 29.93^a,b^	20.71 (−22–69)
Δ% B-MeDM + RW	−12.08 ± 23.20	−8.55 (−50–34)
Δ% B-HFM + RW	−4.97 ± 33.18^b^	−2.05 (−57–81)
Δ% B-MeDM + WW	−7.36 ± 5.66	−9.765 (−11–1)
Δ% B-HFM + WW	−5.37 ± 7.09	−2.755 (−16–1)
Δ% B-MeDM + VDK	−3.37 ± 12.99	−9.765 (−16–27)
Δ% B-HFM + VDK	−2.60 ± 37.38	−2.755 (−101–75)

Results are expressed in mean value ± standard deviation and median, minimum, and maximum for each treatment. Significant values (*p* ≤ 0.05) are expressed as ^a^Δ% B-MeDM versus Δ% B-HFM and ^b^Δ% B-HFM versus Δ% B-HFMRW. B: baseline; RW: fasting red wine; WW: fasting white wine; VDK: fasting vodka; MeDM: Mediterranean meal; HFM: high-fat meal; MeDM + RW: Mediterranean meal plus red wine; MeDM + WW: Mediterranean meal plus white wine; MeDM + VDK: Mediterranean meal plus vodka; HFM + RW: high-fat meal plus red wine; HFM + WW: high-fat meal plus white wine; HFM + VDK: high-fat meal plus vodka.

## Data Availability

The data used to support the findings of this study are available from the corresponding author upon request.

## Abbreviations

ALT/GPT: Alanine transaminase
AST/GOT: Aspartate aminotransferase
AI: Atherogenic index
BAS: Basophiles
CVDs: Cardiovascular diseases
CAT: Catalase
CSI: Cholesterol-saturated fat index
CT: Comparative threshold
CRP: C-reactive protein
EOS: Eosinophils
ESR: Erythrocyte sedimentation rate
FC: Fold change
GPX1: Glutathione peroxidase 1
GRN: Granulocytes
HDL-C: High-density lipoprotein cholesterol
HCT: Hematocrit
HGB: Hemoglobin
HFM: High-fat meal
HOMA-IR: Homeostasis model assessment of insulin resistance
LDL-C: Low-density lipoprotein cholesterol
LYM: Lymphocytes
MCH: Mean corpuscular hemoglobin
MCHC: Mean corpuscular hemoglobin concentration
MCV: Mean corpuscular volume
MPV: Mean platelet volume
MeDM: Mediterranean meal
MON: Monocytes
MUFA: Monounsaturated fatty acids
NO: Nitric oxide
NCDs: Noncommunicable diseases
oxLDL-C: Oxidized LDL-C
PDW: Platelet distribution width
PLT: Platelets
PUFA: Polyunsaturated fatty acids
PRAL: Potential renal acid load
PCT: Procalcitonin
RBC: Red blood cells
RDW: Red cell distribution width
ROS: Reactive oxygen species
RW: Red wine
SOD2: Superoxide dismutase 2
TI: Thrombogenic index
TC: Total cholesterol
Tg: Triglycerides
VDK: Vodka
WBC: White blood cells
WW: White wine
ACTB: *β*-Actin.
